# The predictive effects of individual difference factors on L2 writing complexity, accuracy, and fluency

**DOI:** 10.3389/fpsyg.2025.1631353

**Published:** 2026-01-07

**Authors:** Zihan Gao

**Affiliations:** 1 School of Languages and Communication Studies, Beijing Jiaotong University, Beijing, China; 2 School of Marxism, Beijing Jiaotong University, Beijing, China

**Keywords:** control-value theory, individual-environmental model of writing, individual difference factors, syntactic complexity, lexical complexity, accuracy, fluency

## Abstract

Drawing on the control-value theory and the individual-environmental model of writing, this study investigated the predictive effects of various individual difference factors (i.e., L2 writing grit, enjoyment, anxiety, motivation, and working memory) on L2 writing syntactic complexity, accuracy, lexical complexity, and fluency (CALF) performance. Questionnaires were distributed to 390 high school students to measure their L2 writing grit, enjoyment, anxiety, and motivation. A reading span test and an operation span test were conducted to assess the students' working memory. All participants completed a writing task within 30 minutes. The structural equation modeling results revealed that L2 writing anxiety directly predicted syntactic complexity and accuracy, and L2 writing motivation directly predicted lexical complexity and fluency. Although L2 writing grit did not directly predict CALF performance, it predicted syntactic complexity and accuracy through anxiety. In addition, L2 writing grit predicted lexical complexity and fluency through the serial mediating roles of emotions and motivation. Specifically, the mediating effect of enjoyment and motivation was larger than that of anxiety and motivation in the relationship between grit and lexical complexity as well as between grit and fluency.

## 1 Introduction

L2 writing is a complex and cognitively demanding process that requires learners to actively recall, extract, and integrate relevant knowledge while simultaneously attending to multiple aspects such as content, logic, and language, which draws on learners' cognitive ability and is influenced by various emotional and motivational individual difference (ID) factors (Kormos, 2012; Li et al., 2023). Prior research has mainly examined the effect of cognitive factors (e.g., working memory), motivational factors (e.g., motivation), and negative emotional factors (e.g., anxiety) on L2 writing performance (Guo and Qin, 2010; Lu Y., 2010; Wang, 2021). Inspired by positive psychology, scholarly attention has been increasingly paid to the influence of positive ID factors such as grit and enjoyment as they enhance engagement and counteract the detrimental effect of negative emotions, thereby fostering effective writing process and high-quality output (Zhang and Zhang, 2023; Li et al., 2024). The incorporation of these positive ID factors advances this line of inquiry by providing a more holistic perspective on the role of ID factors in L2 writing performance.

Linguistic features, including L2 writing syntactic complexity, accuracy, lexical complexity, and fluency (CALF), are salient and objective indicators to gauge L2 writing quality (Pun and Li, 2024), trace L2 writing development (Golparvar et al., 2025), and differentiate learners across varying L2 proficiency levels (Barrot and Agdeppa, 2021). These linguistic dimensions compete for the finite attentional resources and ID factors are assumed to influence the sub-dimensions of L2 writing linguistic performance (Skehan and Foster, 2001). As (Skehan and Foster 2001) argue, some learners attempt to use more complex language forms to extend their interlanguage system, while other learners prefer to draw on the familiar linguistic repertoire to ensure accuracy. A multitude of studies concentrated on certain CALF dimensions (Jang and Lee, 2019; Jiang et al., 2025), which may obscure the interrelations between the dimensions of CALF performance and preclude comparisons of how various ID factors differentially predict them. Furthermore, despite the predominant research on the independent effects of ID factors such as anxiety, motivation and working memory on L2 writing CALF performance (Rahimi and Zhang, 2019; Güvendir and Uzun, 2023), there is a scarcity of investigation into positive ID factors (e.g., grit and enjoyment) as well as the interactive effects of various ID factors on L2 writing CALF performance. Therefore, a granular analysis of ID factors and L2 writing CALF performance can not only offer nuanced insights into the relationship between these linguistic dimensions (Liu et al., 2025), but also reveal how varying ID factors jointly influence distinct aspects of linguistic production (Güvendir and Uzun, 2023), thus affording insights of targeted instructional strategies.

The control-value theory (CVT) of achievement emotions proposes the relationship between emotions, their antecedents, and effects (Pekrun, 2006). It assumes that personality factors (e.g., grit) exert effects on emotions (e.g., enjoyment and anxiety) which can further impact academic performance (e.g., L2 writing CALF performance) directly, or influence academic performance indirectly via cognitive factors (e.g., working memory) and motivational factors (e.g., motivation) (Pekrun, 2006; Pekrun et al., 2023). In addition, (Hayes 1996) proposes an individual-environmental model of writing, which underscores cognition and affect in writing. Particularly, it assumes the influence of ID factors, including working memory, motivation, and anxiety in the cognitive processes of writing and written output. The two theories complement each other to underpin the predictive effects of various ID factors on L2 writing. However, there is a lack of empirical research on how these ID factors independently or interactively predict L2 writing CALF performance.

To fill these gaps, the present study aims to examine both the direct and indirect effects^1^ of various kinds of ID factors (i.e., L2 writing grit, enjoyment, anxiety, motivation, and working memory) on L2 writing CALF performance to test the assumptions proposed by the CVT (Pekrun, 2006) and the individual-environmental model of writing (Hayes, 1996), extend their application in the L2 writing context with a specific focus on CALF performance, and elucidate the pathways through which various ID factors predict L2 writing CALF performance.

## 2 Literature review

### 2.1 The impact of L2 writing grit on L2 writing

Grit is defined as the positive personality trait of sustaining efforts and interest to achieve long-term goals despite difficulties or even failure encountered in progress (Duckworth et al., 2007). It has been found to positively correlate with L2 engagement (Sadoughi and Hejazi, 2023), L2 buoyancy (Yang et al., 2024), and language learning performance (Teimouri et al., 2022). Due to the challenges brought about by the intricacy and high cognitive demand inherent in L2 writing, being gritty with deliberate practice is conceived to be a crucial prerequisite in enhancing L2 writing performance (Zabihi, 2018; Zhang and Zhang, 2023). Previous studies on the relationship between grit and L2 writing performance have mainly centered on two branches. On the one hand, observation-based studies examined the role of grit in L2 writing, revealing that gritty students tend to increase feedback seeking behavior (Zhang and Jiang, 2025), enhance cognitive, affective and behavioral engagement in writing (Li and Yuan, 2024), and possess high L2 writing performance (Zhang and Zhang, 2023). On the other hand, intervention-based studies showed that grit intervention significantly increased students' grit level and overall L2 writing performance (i.e., an overall score of indices such as content, organization, and vocabulary) (e.g., Shafiee Rad and Jafarpour, 2023).

Three critical issues warrant further investigation to formulate a better understanding of grit and its role in L2 writing. First, according to (Fathi et al. 2024), grit is skill-specific. For example, learners who are gritty in L2 reading do not necessarily possess the same effort and interest for L2 writing. The measure of L2 writing grit, instead of general grit and L2 grit, is necessary to tap into the kinds of perseverance and interest specific to the L2 writing process. The adoption of L2 writing grit also helps guarantee “local-local consistency” (i.e., the consistency of measuring two constructs at the same level), as reflected in investigating the relationship between L2 writing grit and L2 writing performance (Teimouri et al., 2022, p. 896). Second, previous studies have mainly focused on the direct effect of grit on L2 writing performance (Zhang and Zhang, 2023; Fathi et al., 2024). Despite established associations between grit and emotions (Teimouri et al., 2022), how it interacts with ID factors to jointly predict L2 writing performance remains largely unanswered. Third, although the overall proficiency is practical for assessment, it fails to capture the varying dimensions of performance and their interplay in language development (Skehan and Foster, 2001). CALF indices allow for the precise investigation of how ID factors differentially impact sub-dimensions of linguistic performance, facilitating targeted teaching intervention. Against this background, the present study adopted the measure of L2 writing grit and paid special attention to the CALF indices to untangle the nuanced relationship between L2 writing grit and specific linguistic dimensions, and complement research on the overall L2 writing performance.

### 2.2 The impact of L2 writing anxiety on L2 writing

L2 writing anxiety refers to the disposition of tension and apprehension stimulated by L2 writing (Cheng, 2004; Güvendir and Uzun, 2023). As a multi-dimensional construct, L2 writing anxiety can be conceptualized as cognitive anxiety, somatic anxiety, and avoidance behavior (Cheng, 2004), which arises from various internal and external factors such as teacher feedback, conceiving difficulty, and lack of confidence (Guo and Qin, 2010). This negative emotion has been widely examined in relation to the overall writing performance, with studies generally reporting negative associations (Guo and Qin, 2010; Wang, 2021). Anxious students, due to their low engagement (Wang et al., 2024a) as well as insufficient use of self-regulation strategies (Teng and Pan, 2024), are likely to achieve unsatisfactory L2 writing performance. However, its effect, especially the interplay with other ID factors on L2 writing CALF performance remains unclear.

Existing studies have examined the relationship between L2 writing anxiety and linguistic performance (e.g., Zabihi, 2018). (Zabihi 2018) revealed that L2 writing anxiety negatively predicted L2 writing syntactic complexity, accuracy, and fluency in the narrative writing. This relationship is further explored across different genres (Zabihi et al., 2020) as well as task complexity conditions manipulated along the resource directing dimension via the number of task elements and reasoning demands (Rahimi and Zhang, 2019; Abdi Tabari and Goetze, 2024; Abdi Tabari et al., 2025), or along the resource dispersing dimension via planning time, the number of tasks and steps, and task structure (Güvendir and Uzun, 2023). For example, (Zabihi et al. 2020) showed that L2 writing anxiety exerted more extensive effects on linguistic performance in the argumentative writing compared with narrative writing. Specifically, somatic anxiety and cognitive anxiety were only negatively related to accuracy in the narrative writing, while cognitive anxiety was negatively correlated with syntactic complexity, accuracy and fluency, and avoidance behavior was negatively associated with syntactic complexity and accuracy in the argumentative writing.

As for the relationship under different task complexity conditions, results remained mixed. Among various measures of complexity, accuracy, and fluency, (Abdi Tabari and Goetze 2024) found that the overall L2 writing anxiety was only significantly and negatively related to lexical complexity in the simple argumentative task rather than the complex one among high L2 proficiency learners. However, (Rahimi and Zhang 2019) revealed that L2 writing anxiety measured by avoidance behavior was only significantly related to syntactic complexity in the complex argumentative writing task instead of the simple one among upper-intermediate L2 proficiency learners. The discrepency can be explained by the different measurements of L2 writing anxiety and learners' varying L2 proficiency levels. (Güvendir and Uzun 2023) extended prior research by examining the mediating effect of working memory, indicating that anxiety impaired working memory which in turn constrained syntactic complexity. While this finding provides important initial evidence for cognitive mediation in L2 writing, it focused exclusively on syntactic complexity. The present study addresses the gaps by further examining the direct relationship between anxiety and CALF performance, and more importantly investigating how anxiety indirectly influences the diversifying CALF measures.

### 2.3 The impact of L2 writing enjoyment on L2 writing

L2 writing enjoyment is a frequently experienced positive activating emotion evoked by L2 writing (Li et al., 2023). Given its prevalence and facilitative role in enhancing motivation and buoyancy (Wang and Xu, 2024) as well as improving the use of self-regulated writing strategies (Wang et al., 2024b), researchers have increasingly attended to its effect on the overall L2 writing performance (Zhu et al., 2022; Zhao et al., 2023; Li et al., 2024). Recent studies have yielded mixed findings regarding the relationship between L2 writing enjoyment and L2 writing performance, indicating significant positive associations (Zhu et al., 2022) or non-significant ones (Tahmouresi and Papi, 2021; Zhao et al., 2023; Yao et al., 2024). The educational level is one possible explanation of the discrepancy, as high school students in (Yao et al. 2024) experienced lower L2 writing enjoyment compared with the undergraduate students in (Zhu et al. 2022) due to the pressure caused by the college entrance examination, leading to the non-significant association between L2 writing enjoyment and writing performance. The discrepancy can also be explained by the possible mediating role of motivated learning behavior (Tahmouresi and Papi, 2021) or L2 writing strategies (Zhao et al., 2023) which mitigated the predictive effect of L2 writing enjoyment on writing performance.

Moving beyond the global performance measure, (Wu and Halim 2024) and (Abdi Tabari et al. 2024) were among the first attempts to investigate the relationship between L2 writing enjoyment and CALF performance. (Wu and Halim 2024) examined the interactive effect of task complexity and L2 writing enjoyment on CALF performance. Results showed that students with high L2 writing enjoyment produced a higher level of syntactic complexity in the complex writing task than the simple writing task, while students with low L2 writing enjoyment achieved a higher level of syntactic complexity in the simple writing task than the complex writing task. (Abdi Tabari et al. 2024) conducted a longitudinal investigation into the association between enjoyment and CALF performance of decision-making writing tasks. The findings indicated that L2 writing enjoyment only positively correlated with syntactic complexity at the beginning of the semester in stage 1, while it positively linked to almost all the CALF measures in stage 2, and the magnitude of the significant relationship generally increased in stage 3. Despite the significance, both studies focused solely on undergraduate students, whose enjoyment may differ from that of high school students due to various individual and environmental factors such as trait emotional intelligence and pressure from critical exams (Li et al., 2021; Yao et al., 2024). More empirical research is needed to further reveal the relationship between L2 writing enjoyment and CALF performance in different educational levels to allow for nuanced comparisons and draw conclusive insights.

### 2.4 The impact of L2 writing motivation on L2 writing

L2 motivation affords the fundamental impetus to initiate and sustain the L2 learning process (Dörnyei, 2005). Considering the complex cognitive demands imposed by L2 writing, investigating motivation is of paramount importance, as it serves to enhance learning engagement in writing (Wang et al., 2025) and improve self-regulated learning (Wang et al., 2024b). Motivation can be conceptualized from different perspectives such as ideal L2 self and ought-to L2 self (Dörnyei, 2005) as well as intrinsic and extrinsic motivation (Pintrich et al., 1991). The constructs of intrinsic motivation and extrinsic motivation in L2 writing were adopted for the following two reasons. First, intrinsic motivation (e.g., learning interest) and extrinsic motivation (e.g., desire to achieve goals) resonate with tenets in positive psychology which argue that agentic individuals can perform actions due to internal or external stimuli rather than passively accepting a reality (Seligman and Csikszentmihalyi, 2000). Intrinsic and extrinsic writing motivation were revealed to be positively correlated with each other (Pintrich et al., 1991), which can collectively constitute the higher order construct of motivation (Gan et al., 2023; Graham et al., 2023). Second, previous studies adopting the writing motivation questionnaire based on intrinsic motivation and extrinsic motivation have shown satisfactory reliability and predictive effects on L2 writing strategies (Teng, 2024).

Empirical research on the impact of L2 writing motivation on linguistic features often concentrated on a limited subset of CALF dimensions, including syntactic and lexical complexity (Zalbidea, 2024), syntactic complexity and accuracy (Rahimi and Zhang, 2019), as well as fluency (Jang and Lee, 2019). For example, (Rahimi and Zhang 2019) revealed that L2 writing motivational beliefs significantly correlated with syntactic complexity and accuracy to a larger extent in the complex task than the simple one. (Jang and Lee 2019) found a significant positive correlation between the ideal L2 self and fluency. Departing from the aforementioned studies, (Farahani et al. 2020) adopted a qualitative approach to examine various dimensions of linguistic performance, including L2 writing syntactic complexity, accuracy, and fluency and their interaction with motivation through tracing one L2 learner over a year. Results revealed that motivation, syntactic complexity, and fluency followed similar trajectories, while the pattern of accuracy was generally in an opposite direction. In other words, with higher motivation, the learner was likely to produce more complex, more fluent but less accurate writing output. As highlighted by (Skehan and Foster 2001), the limited attentional capacity imposes competing demands during language production, wherein prioritizing one linguistic dimension (e.g., complexity) often results in trade-offs in others (e.g., accuracy). By further exploring the relationship between motivation and all the L2 writing linguistic dimensions with a quantitative approach, it is possible to verify a general pattern concerning whether motivation differentially prioritizes specific CALF dimensions or enables synergistic development across the dimensions.

### 2.5 The impact of working memory on L2 writing

Working memory, a limited capacity system responsible for temporarily storing and manipulating information (Baddeley, 2003), is involved in every stage (e.g., planning, translation, execution, and monitoring) of L2 writing (Kormos, 2012). Empirical studies have examined the relationship between working memory and L2 writing CALF performance, yielding mixed results (Cho, 2018; Zabihi, 2018; Vasylets and Marín, 2021; Son, 2022; Manchón et al., 2023; Jiang et al., 2025). The discrepancy among these studies may be attributed to various methodological factors such as different measurements of working memory, the language stimuli of working memory test and task genre. Specifically, working memory measured by operation span test was found to positively predict syntactic complexity and fluency (Zabihi, 2018) and high working memory assessed by reading span test was also associated with high L2 writing accuracy (Vasylets and Marín, 2021), while (Manchón et al. 2023) showed that working memory evaluated by *n*-back test was not significantly correlated with any measure of CALF performance. As mentioned by (Granena 2023), *n*-back test emphasizes familiarity and recognition-based cognitive process, while complex span tasks such as the operation and reading span tests highlight active recall process, which may explain the different results in these studies. Language stimuli (i.e., L1 or L2 working memory tests) can also affect the relationship between working memory and L2 writing linguistic performance even when studies both used reading span tests (Cho, 2018; Vasylets and Marín, 2021). In a systematic review concerning their relationship, (Li 2023) recommended the use of L1 instead of L2 working memory test, on the grounds that the latter is “subject to learners' L2 proficiency” (p. 22). Genre is another potential moderator since different genres necessitate distinct features to achieve communicative functions (Yoon and Polio, 2017; Zabihi et al., 2020). Argumentative writing is generally more cognitively-demanding than narrative writing, as it requires reliable evidence and persuasive reasoning to construct a solid argument (Zabihi et al., 2020). Linguistically, the heightened cognitive demand is reflected in features such as higher levels of length of production unit, phrasal complexity, and lexical sophistication in the argumentative writing compared to narratives (Yoon and Polio, 2017). Given these demands, both in content construction and linguistic expression, argumentative writing may impose greater cognitive load on working memory, leading it to exert larger effects on linguistic performance in this genre.

Additionally, previous studies mainly investigated the independent effect of working memory on L2 writing CALF performance under different task conditions such as varying task types (Jiang et al., 2025) and task complexity (Son, 2022). (Zabihi 2018) further attended to the combined effects of working memory, and anxiety on L2 writing complexity, accuracy, and fluency, revealing that both ID factors significantly predicted L2 writing linguistic performance. However, the interactive effects of working memory and emotions on L2 writing CALF performance remains underexplored and prior studies have largely confined emotions to anxiety (Güvendir and Uzun, 2023). To further elaborate the cognition-emotion interplay in shaping L2 writing performance (Li et al., 2024), it is crucial to examine the mediating role of working memory in the relationship between various emotions and L2 writing CALF performance, thereby providing deep insights into the psychological underpinnings and cognitive processes.

### 2.6 Associations between ID factors

Previous studies have also attended to the relationships between these ID factors. Regarding the association between grit and emotions, prior research was mainly conducted in the general L2 learning context, showing that L2 grit positively correlated with L2 enjoyment and negatively associated with L2 anxiety (Teimouri et al., 2022; Zhao et al., 2024). Moving into the specific skill of L2 writing, the negative association was consistent between L2 writing grit and L2 writing anxiety, as gritty learners tend to be resilient, determined, and confident to enhance abilities which help alleviate their anxiety (Zhang and Jiang, 2025). L2 writing emotions exerted differential effects on writing motivation, with enjoyment positively associating with motivation (Li et al., 2024) and anxiety negatively relating to motivation (Wang, 2021; Wang and Xu, 2024). As for the link between emotions and working memory, several studies found that positive emotions positively associated with working memory and negative emotions negatively related to working memory (Storbeck and Maswood, 2016; Güvendir and Uzun, 2023), while non-significant links were revealed in (Simard et al. 2025). More empirical research is necessitated to formulate a clear picture concerning emotions and working memory. In addition, despite these associations between ID factors, the interactive effects of various ID factors on L2 writing CALF performance warrant further investigation.

### 2.7 The hypothesized model

The CVT (Pekrun, 2006) and the individual-environmental model of writing (Hayes, 1996) were integrated to hypothesize the direct and indirect effects of ID factors (i.e., L2 writing grit, enjoyment, anxiety, motivation, and working memory) on L2 writing CALF performance. The CVT hypothesizes the direct effects of various ID factors, including personality traits, emotions, motivation, and working memory on academic performance (Pekrun, 2006). Specifically, positive personality traits (e.g., grit) are assumed to be positively linked to academic performance (Pekrun et al., 2023). Emotions are at the core of the CVT, which are reflected as trait emotions (i.e., habitual emotions accumulated over a long period of time) and state emotions (i.e., momentary emotions occurring at a certain point of time) (Pekrun, 2006). The repeated recurrence of state emotions in a specific situation can give rise to trait emotions, which predispose learners to experience certain emotions whenever they enter that situation and exert lingering effects on academic performance. Positive emotions (e.g., enjoyment) are assumed to be positively associated with academic performance, while negative emotions (e.g., anxiety) negatively related to academic achievement (Pekrun et al., 2023). In addition, working memory represents a vital cognitive resource for memory storage and processing and acts as a prerequisite for successful task completion, which is expected to positively correlate with academic performance (Pekrun and Linnenbrink-Garcia, 2012). Motivation enables students to engage in learning, thus associating with high academic achievement (Pekrun et al., 2023). Particularly in writing, (Hayes 1996) assumes that ID factors, including working memory, motivation, and anxiety can directly influence the cognitive processes underlying writing. According to the individual-environmental model of writing (Hayes, 1996), working memory occupies a central position, functioning to store, process, and retrieve relevant information as well as allocate cognitive resources during the writing process. When learners' knowledge has not yet reached an automatic level, they rely on working memory to perform these functions during writing (Hayes and Chenoweth, 2006). Motivation, as a goal-directed willingness, enhances writing engagement and effective learning strategy selection, which is assumed to positively influence writing performance. Anxiety undermines learners' confidence in their own abilities, leading them to reduce engagement in text interpretation, reflection, and production, thereby negatively impacting their writing performance.

As for the indirect effects, the CVT emphasizes the mediating roles of emotions in the relationship between personality traits and academic achievement (Pekrun et al., 2023). Students with positive personality traits tend to positively appraise their competence and perceive high intrinsic value in learning activities and outcomes, leading them to experience more enjoyment and less anxiety, which contributes to their higher academic performance. In addition, the CVT posits the serial mediating effects of emotions as well as cognitive and motivational factors on the relationship between personality traits and academic achievement (Pekrun, 2006; Pekrun et al., 2023). Personality traits exert effects on emotions, and positive emotions are thought to facilitate attentional engagement with the task, which in turn, allows learners to allocate more working memory resources to complete the task, thus promoting academic achievement. Negative emotions, on the contrary, may invoke task-irrelevant thinking, which reduces the working memory resources for task completion, and impairs the efficiency of cognitive processing, thereby hindering academic achievement (Pekrun and Linnenbrink-Garcia, 2012). Additionally, positive emotions strengthen intrinsic and extrinsic motivation to learn, which is facilitative for academic achievement, while negative emotions impair intrinsic motivation, which may debilitate learning performance (Pekrun, 2006). The individual-environmental model of writing particularly proposes the mediating role of working memory in the relationship between anxiety and writing performance (Hayes, 1996). Specifically, anxiety tends to reduce learners' cognitive and behavioral engagement during text interpretation, reflection, and production, thus decreasing the cognitive resources that should be allocated to the writing task. This is detrimental to working memory's capacity to process and retrieve topic-related content and relevant linguistic knowledge, ultimately degrading writing performance.

While previous research investigated the direct effects of ID factors, including L2 writing anxiety, motivation, and working memory on L2 writing CALF performance (Rahimi and Zhang, 2019; Manchón et al., 2023), mixed results existed and the investigation of a limited subset of CALF dimensions precluded a comprehensive understanding of the interrelationships. Besides, scant attention has been paid to the predictive effects of L2 writing grit and enjoyment on CALF performance. In addition, according to the CVT and the individual-environmental model of writing, personality antecedents can predict writing performance through emotions or through the serial mediating effects^2^ of emotions as well as cognitive and motivational factors. However, empirical evidence that demonstrates the mediating effects of these ID factors is scarce.

**Figure 1:**
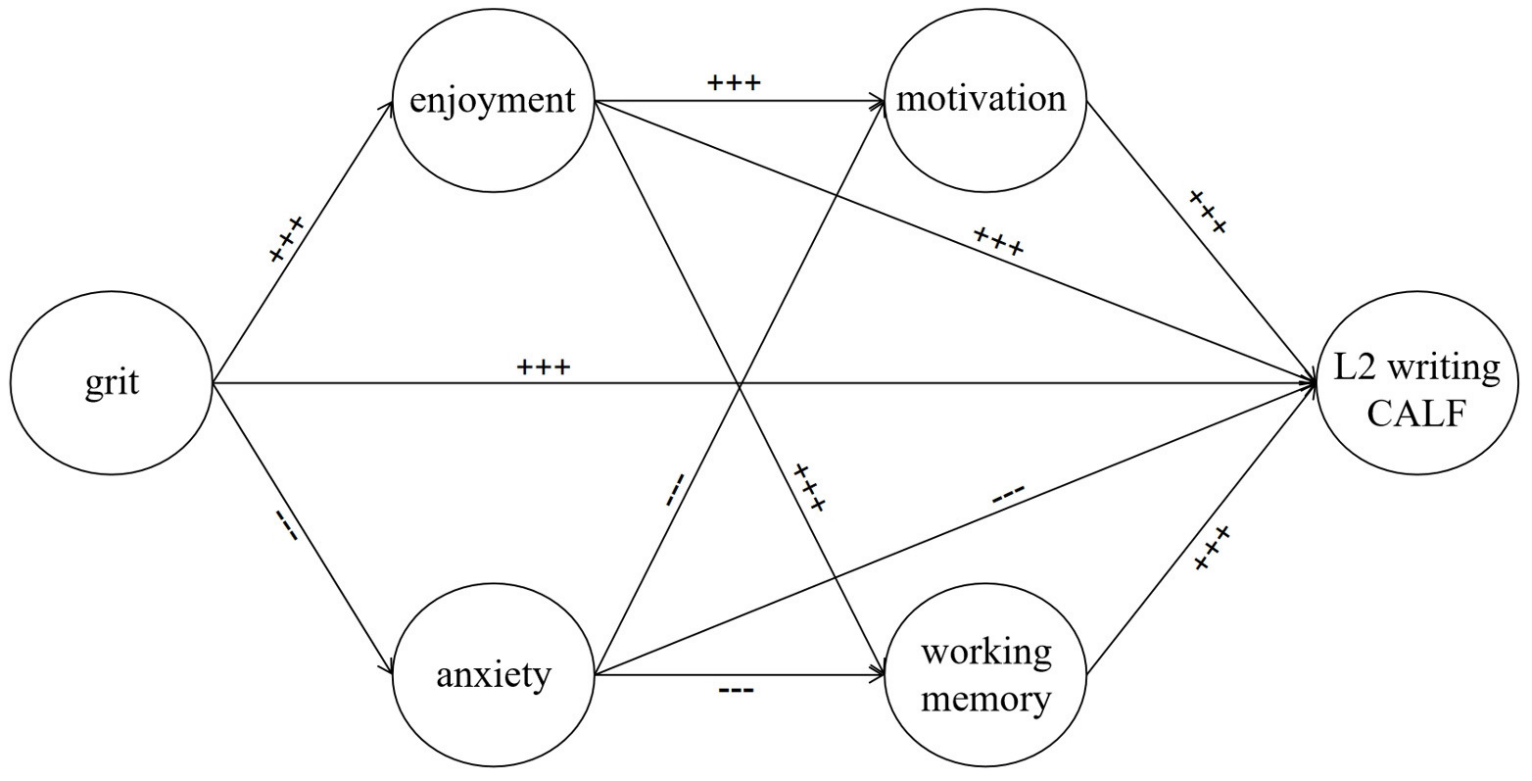
The hypothesized model of ID factors and L2 writing CALF performance. − − − = expected negative path; + + + = expected positive path.

Therefore, the present study aimed to answer the following two research questions (RQ):

RQ1: What are the direct effects of ID factors (i.e., L2 writing grit, enjoyment, anxiety, motivation, and working memory) on L2 writing CALF performance? RQ2: What are the indirect effects of L2 writing grit on L2 writing CALF performance via emotional, cognitive, and motivational factors? Based on the CVT, the individual-environmental model of writing, and relevant empirical studies, the hypothesized model is demonstrated in Figure 1, and hypotheses (Hs) are proposed. H1a L2 writing grit positively predicts L2 writing CALF performance. H1b L2 writing enjoyment positively predicts L2 writing CALF performance. H1c L2 writing anxiety negatively predicts L2 writing CALF performance. H1d L2 writing motivation positively predicts L2 writing CALF performance. H1e Working memory positively predicts L2 writing CALF performance. H2a L2 writing grit predicts L2 writing CALF performance indirectly through enjoyment. H2b L2 writing grit predicts L2 writing CALF performance indirectly through anxiety. H2c L2 writing grit predicts L2 writing CALF performance indirectly through enjoyment and motivation. H2d L2 writing grit predicts L2 writing CALF performance indirectly through enjoyment and working memory. H2e L2 writing grit predicts L2 writing CALF performance indirectly through anxiety and motivation. H2f L2 writing grit predicts L2 writing CALF performance indirectly through anxiety and working memory.

## 3 Methodology

### 3.1 Participants

Convenience sampling was adopted in the present study. Three hundred and ninety students from a senior high school in northern China voluntarily participated in the study. Consent forms were obtained from the participants, their guardians, school administrators, and their teachers. The students spoke Mandarin Chinese as mother tongue and they attended five English classes (each lasting 45 min) every week. The English classes included activities related to sub-skills of English. English writing in each unit was taught in two to three lessons in which the teacher instructed on content, organization, and linguistic usage of narrative, argumentative, or expository writing. The instructor provided feedback on the students' writing assignments.

After initial screening for subjects providing careless answers and outliers, the final sample consisted of 348 students^3^. The cohort comprised 167 male students (47.99%) and 181 female students (52.01%), ranging in age from 15 to 18, with an average age of 15.85 (*SD* = 0.56). The participants had studied English for 7.90 years on average (*SD* = 0.73, observed range = 5–13). The average score of the final English exam was 100.84 out of 150 (*SD* = 16.40, observed range = 50–136) and the average self-perceived English proficiency was 5.34 out of 10 (*SD* = 1.85, observed range = 1–10), indicating their lower-intermediate level of English proficiency. Based on the median of final English exam score (Median = 102), the participants were classified into low proficiency group (*N* = 176) and high proficiency group (*N* = 172). Independent-samples *t*-tests revealed that they differed significantly in various ID factors, including enjoyment, *t*_(346)_ = −4.981, *p* = 0.000, anxiety, *t*_(346)_ = 2.826, *p* = 0.005, motivation, *t*_(346)_ = −4.315, *p* = 0.000, and working memory, *t*_(346)_ = −3.277, *p* = 0.001. The indicators above generally showed the relative heterogeneity across participants' ID factors as well as English learning experience and achievement.

### 3.2 Instruments

A composite 48-item questionnaire with four scales was administered to evaluate the participants' L2 writing grit, anxiety, enjoyment, and motivation (See Supplementary material). The participants responded to these seven-point Likert scales ranging from 1 (i.e., completely disagree) to 7 (i.e., completely agree). The Chinese versions of the scales were implemented to ensure participants' accurate comprehension of the items. For the scales originally written in English (i.e., grit and motivation), translation and back translation were performed and inconsistencies between the Chinese version and the English version were resolved. The questionnaire was piloted among 35 students of the same proficiency level. Items with poor readability were refined to ensure participants' full understanding of the questionnaire.

#### 3.2.1 L2 writing grit scale

The L2 writing grit scale was adapted from the L2 grit scale (Teimouri et al., 2022) by adding elements particular to L2 writing to meet the needs of the present study, which has demonstrated high reliability with Cronbach's alpha being 0.824 in (Fathi et al. 2024). The L2 writing grit scale consists of 5 items measuring “perseverance of effort” (e.g., “When it comes to English writing, I am a hard-working learner.”) and 4 items evaluating “consistency of interest” (e.g., “My interests in learning English writing change from year to year.”). Confirmatory factor analysis (CFA) results verified an acceptable model fit of the L2 writing grit scale in the present study (*x*^2^*/df* = 2.791; CFI = 0.964; TLI = 0.951; SRMR = 0.036; RMSEA = 0.070) with factor loadings ranging from 0.678 to 0.798 (Hair et al., 2014). The reliability of the overall scale was good with Cronbach's alpha being 0.861.

#### 3.2.2 L2 writing anxiety scale

The foreign language writing anxiety scale was used to assess participants' L2 writing anxiety (Guo and Qin, 2010). The 20-item foreign language writing anxiety scale is divided into four sub-dimensions: classroom teaching anxiety (5 items; e.g., “I am afraid that my English essay will be chosen for class discussion or evaluation.”), conceiving anxiety (5 items; e.g., “I can't think clearly when I write an English essay within a time limit.”), avoidance behavior (6 items; e.g., “I usually avoid writing essays in English whenever possible.”) and lack of confidence (4 items; e.g., “I worry about getting a low mark when my English essay is to be corrected.”). The reliability of this scale in the Chinese context was high, with Cronbach's alpha being 0.842 (Guo and Qin, 2010). In the present study, the model fit of the scale was satisfactory via CFA (*x*^2^*/df* = 1.540; CFI = 0.962; TLI = 0.957; SRMR = 0.039; RMSEA = 0.038) and the reliability was relatively high (Cronbach's alpha = 0.896). The factor loadings fell between 0.554 and 0.862.

#### 3.2.3 L2 writing enjoyment scale

The L2 writing enjoyment scale was adapted from the Chinese version of the foreign language enjoyment scale which exhibited good reliability in the Chinese context (Cronbach's alpha = 0.826) (Li et al., 2018). In order to suit the purpose of the present study, items related to “English” in the original scale were modified into “English writing.” The adapted scale comprises 5 items for FLE-private (e.g., “Learning English writing is fun.”), 3 items for FLE-teacher (e.g., “The English writing teacher is friendly.”) and 3 items for FLE-atmosphere (e.g., “We form a tight English writing group.”). In the present study, CFA results indicated that the scale showed good model fit (*x*^2^*/df* = 3.101; CFI = 0.958; TLI = 0.944; SRMR = 0.039; RMSEA = 0.075) with factor loadings falling between 0.725 and 0.869. The reliability in the present study was satisfactory with Cronbach's alpha being 0.892.

#### 3.2.4 L2 writing motivation scale

The L2 writing motivation scale was adapted from the motivation questionnaire developed by (Pintrich et al. 1991) to specify academic situations into L2 writing situations. The adapted version of the questionnaire contains four items that are used to assess intrinsic writing motivation (e.g., “I prefer English writing course material that arouses my curiosity, even if it is difficult to learn.”) and another four items for evaluating extrinsic writing motivation (e.g., “Getting a good grade in English writing is satisfying for me.”). This adapted scale has previously been used to measure Chinese students' writing motivation, demonstrating good reliability for intrinsic writing motivation (Cronbach's alpha = 0.83) and extrinsic writing motivation (Cronbach's alpha = 0.81) (Teng, 2024). Given the distinct yet complementary nature of intrinsic and extrinsic motivation (Pintrich et al., 1991; Graham et al., 2023), a two-factor second-order model of motivation was specified to represent the overarching motivation construct. The CFA results of the adapted scale in the present study indicated acceptable model fit (*x*^2^*/df* = 3.051; CFI = 0.975; TLI = 0.963; SRMR = 0.036; RMSEA = 0.075), with factor loadings ranging from 0.666 to 0.885. The adapted scale showed high reliability with Cronbach's alpha being 0.876 in the present study.

#### 3.2.5 Working memory tests

The present study used two complex span working memory measures to assess the storage and processing components of working memory (Baddeley, 2003), following the suggestion of (Conway et al. 2005) which posited that no single working memory test can perfectly assess working memory since each test taps into different abilities. The Chinese versions of both working memory tests (i.e., L1 WM tests) were adopted for two reasons. First, the results of L2 WM tests can be influenced by learners' L2 proficiency, which may lead to a confounding effect of L2 proficiency and WM on language performance, thereby inflating the WM-L2 outcome correlation estimate (Linck et al., 2014). As suggested in a meta-analysis of WM and L2 comprehension and production (Linck et al., 2014), L1 working memory tests should be adopted if a study aims to “purely measure WM” and “isolate the true relationship between WM and L2 proficiency” (p. 872), which suits the present study. Second, in a systematic review of the association between working memory and L2 writing, (Li 2023) found that among the 16 studies he reviewed, most of the studies (i.e., 11 studies) adopted L1 working memory tests. The adoption of L1 working memory tests in the present study can therefore help maintain cross-study consistency, facilitating the synthesis of findings in the relationship between working memory and L2 writing performance.

In the present study, a modified version of the reading span test described by (Daneman and Carpenter 1980), adopted by (Dallas 2004), was selected to measure participants' working memory. Participants were required to perform the dual tasks of making true-false judgement on 15 sets of sentences ranging from 2 to 6 in each set and memorizing the final words of the sentences (i.e., two Chinese characters). The final score of the reading span test was calculated by adding the correct judgement score and memorization score.

The operation span test was similar to the reading span test except that the sentences were all replaced into strings of mathematical operations and words (Turner and Engle, 1989). The adapted version of Lu Y. (2010) was used to assess working memory. The average of the reading span and operation span tests scores constituted the participants' working memory score. Twenty percent of the tests were randomly selected to be assessed by another ratter who was given the answer key and trained in the scoring protocol through detailed explanation and a practice rating session. The working memory tests comprised objective and unambiguous answers, including the judgement of true or false choice and a series of two Chinese characters. The two sets of scores were completely consistent, resulting in the inter-rater reliability coefficient of 1.

#### 3.2.6 Writing task

All of the participants were required to write at least 150 words within 30 min without referring to any materials on one of the two writing tasks^4^ in the retired version of the Matriculation English Test in the National College Entrance Examination which is a nationwide high-stake examination in China with equivalent test contents. A random selection of half number of students wrote an essay on Topic 1, introducing a person and explaining why he/she deserved respect, while the other half wrote an essay on Topic 2, explaining his/her advantages of being a volunteer and the things he/she could do as a volunteer. This random allocation helped ensure that any pre-existing differences in learners' ID factors or writing ability were equally distributed across the two topics, thereby minimizing the potential bias of topic selection. Independent-samples *t*-tests verified that the two topic groups did not differ significantly on any ID factor, including grit [*t*_(368)_ = −0.921, *p* = 0.358], enjoyment [*t*_(368)_ = 0.071, *p* = 0.943], anxiety [*t*_(368)_ = −1.871, *p* = 0.062], motivation [*t*_(368)_ = −1.283, *p* = 0.200], and working memory [*t*_(368)_ = 1.277, *p* = 0.202]. Furthermore, moderation analyses showed that all Topic × ID interaction terms were non-significant across the CALF measures (*p*s > 0.05), indicating that topic did not moderate the effect of ID factors on L2 writing CALF performance. Forty-eight students of the same proficiency level who did not participate in the main study were invited to rate the level of difficulty and familiarity of the two topics from 1 (i.e., not difficult/familiar at all) to 5 (i.e., very difficult/familiar). Paired-samples *t*-test revealed no significant difference between the two topics in terms of difficulty, *t*_(47)_ = −1.279, *p* = 0.207, and familiarity, *t*_(47)_ = 1.656, *p* = 0.104. In the pilot study, the perceived difficulty ratings from 1 to 5 for Topic 1 were 6.3%, 20.8%, 35.4%, 27.1%, and 10.4%, respectively, and for Topic 2 were 6.3%, 14.6%, 33.3%, 27.1%, and 18.8%, respectively, verifying the variability of students' perceptions of the topic difficulty. In the main study, the participants' perceived difficulty of Topic 1 (*M* = 3.146, *SD* = 1.072) and Topic 2 (*M* = 3.375, *SD* = 1.142) exceeded the midpoint of 3, suggesting that they generally found the tasks moderately challenging.

### 3.3 Data collection

**Figure 2:**
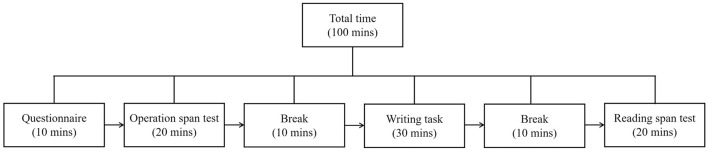
Data collection procedure.

The participants were required to complete the questionnaires, the working memory tests, and the writing task in their classroom within two class periods with an English teacher and a head teacher supervising the process. All the tests were in a paper-and-pencil format. Participants were asked to complete the questionnaires within 10 minutes and the operation span test within 20 minutes. After a 10-minute break, the students finished the writing task independently within 30 minutes. After another 10-minute break, the participants finally completed the reading span test within 20 minutes. The data collection procedure is presented in Figure 2.

The sequencing of the data collection followed both theoretical and practical considerations. First, the questionnaires were administered at the beginning because constructs such as grit, motivation, and affective dispositions are theorized as trait-like antecedents (Dörnyei, 2005), which can be collected before working memory tests (Zhang and Zhang, 2023) and have shown their roles in predicting L2 writing performance (Rahimi and Zhang, 2019; Zabihi et al., 2020). Second, the two working memory tests were separated by the writing activity, as working memory is a capacity-limited system that becomes less efficient when heavily taxed (Baddeley, 2003). Distributing them helped avoid cognitive fatigue and preserved more authentic performance on both working memory tests. Third, tasks were deliberately interspersed with 10-min breaks and varied task types to reduce fatigue and monotony. The answers to the questionnaire items and the scores of working memory tests were computerized into SPSS. The writing data were transcribed into electronic texts in Microsoft Word with careful word-by-word examination.

### 3.4 Data analysis

#### 3.4.1 Measures of L2 writing performance

The present study selected the commonly adopted metrics for L2 writing CALF (See Table 1) to enable comparison with previous research findings. Indices of syntactic complexity should cover different subdimensions, each of which needs no more than one specific measurement to gauge distinctness and avoid redundancy (Norris and Ortega, 2009). Therefore, the study selected four indices (i.e., MLT, C/T, CN/T, CP/T) across four major subdimensions for syntactic complexity (Lu X., 2010; Zhan et al., 2024). L2 syntactic complexity analyzer (Lu X., 2010) was used to measure the above indices.

**Table 1:** L2 writing CALF measures in the present study.

**Construct**	**Sub-construct**	**Indices**	**Instrument**
Syntactic complexity	Length of production unit	Mean length of T-unit (MLT)	L2 syntactic complexity analyzer (Lu X., 2010)
	Subordination complexity	Clauses per T-unit (C/T)	
	Phrase complexity	Complex nominals per T-unit (CN/T)	
	Coordination complexity	Coordinate phrases per T-unit (CP/T)	
Lexical complexity	Lexical density	Lexical words/words (LD)	L2 lexical complexity analyzer (Lu, 2012)
	Lexical variation	Corrected type-token ratio (CTTR)	
	Lexical sophistication	Lexical sophistication-II (LS2)	
Accuracy	Error-free ratio	Error-free T-units/T-units (EFT/T)	Manual coding
		Error-free clauses/clauses (EFC/C)	Grammarly
Fluency	Number	Number of words (W)	L2 syntactic complexity analyzer (Lu X., 2010)
		Number of T-units (T)	
		Number of clauses (C)	

Lexical complexity was assessed by lexical density, lexical variation, and lexical sophistication via L2 lexical complexity analyzer (Lu, 2012). The selected indices of LD, CTTR and LS2 have been revealed to be significantly correlated with L2 writing performance (Yang et al., 2023).

Accuracy was measured by the ratios of error-free T-units and clauses (Zabihi et al., 2020). Lexical and syntactic errors were considered errors, while capitalization and punctuation errors were excluded (Zabihi, 2018; Panagopoulos et al., 2024). Errors were identified manually for all the writing texts, and Grammarly was used to automatically identify errors in 20% of the randomly selected writing texts. The inter-rater reliability was relatively high for EFT/T (*r* = 0.911) and EFC/C (*r* = 0.880). Therefore, the results of manual identification were used to evaluate accuracy.

Finally, the number of words was adopted to measure fluency since it was found to be a reliable predictor of fluency in writing within a consistent and limited time (Abdi Tabari and Goetze, 2024). Following (Zabihi et al. 2020), fluency was also assessed by the number of T-units and clauses.

#### 3.4.2 Statistical analyses

Descriptive analysis, normality, correlational analysis, multicollinearity, and the reliability of the scales were conducted using SPSS 26 software. The construct validity of the measurement models of the four scales was examined via CFA using AMOS 26. The follow-up analyses adopted a confirmatory approach via structural equation modeling (SEM) to evaluate whether the theoretically specified pattern of relations aligned with the data. Item parceling was adopted to reduce the item-level measurement errors and obtain a more favorable parameter-to-sample size ratio, thereby achieving more reliable results for the complex SEM model (Little et al., 2002). Parcels were constructed based on the conceptual facets of each scale, as CFA revealed acceptable construct validity of the multi-factor second-order models. Power analysis was conducted to estimate the minimum sample size required for the SEM (Soper, 2013). Results revealed that at least 232 participants were needed to detect a medium effect size of 0.30 with a statistical power of 0.80, given five latent variables and 14 observed variables. In addition, the parameter-to-sample size ratio was examined. According to (Bentler and Chou 1987), a minimum ratio of 1:5 is required under normal distribution assumptions. As the present model included 38 free parameters, the minimum sample size required was 190. Taken together, both the power analysis and parameter-to-sample size criterion indicated that the sample size of the present study was adequate for SEM estimation. A model is considered acceptable when the thresholds of these indicators are verified (*x*^2^*/df* < 3, CFI > 0.90, TLI > 0.90, SRMR < 0.07, RMSEA < 0.08) (Hair et al., 2014). Direct effect sizes are measured through standardized regression weighs based on Cohen et al's. (2018) suggestion of weak effect size (0.00 < β < 0.10), modest effect size (0.10 < β < 0.30), moderate effect size (0.30 < β < 0.50), and strong effect size (β > 0.50). The mediating effect is tested by conducting the Bootstrap technique with 5,000 repeated samples to generate 95% confidence intervals, and the mediating effect is considered significant if zero is not included in the 95% confidence intervals (Hayes, 2018). According to (Fritz and MacKinnon 2007), 0.02, 0.05, 0.15, and 0.35 are regarded as very small, small, medium, and large mediating effect sizes, respectively.

## 4 Results

### 4.1 Preliminary analyses

Descriptive statistics as shown in Table 2 demonstrates that the students possess a medium level of L2 writing grit, enjoyment, anxiety as well as a high level of motivation and working memory. The skewness and kurtosis of these variables fell between −2 and 2, indicating normal distribution (Kunnan, 1998). The inter-relationship between the ID factors as well as between ID factors and L2 writing performance ranged from −0.241 to 0.516, lower than the multicollinearity threshold of 0.80 (Field, 2013). Values of Tolerance and Variance Inflation Factors were also checked with the former above 0.10 and the latter below 2, indicating the lack of multicollinearity (Kline, 2016).

**Table 2:** Descriptive statistics of the ID variables.

**Variables**	**Mean**	***SD***	**Min**	**Max**	**Skewness**	**Kurtosis**
Grit	4.62	1.06	2.22	7.00	0.05	−0.91
Enjoyment	4.50	1.02	1.91	6.55	−0.03	−0.91
Anxiety	4.79	0.96	2.90	6.70	0.06	−0.98
Motivation	5.16	1.09	1.88	7.00	−0.37	−0.58
Working memory	108.52	6.83	87.50	119.00	−1.07	0.87

Table 3 shows the descriptive statistics of students' L2 writing CALF performance, which was consistent with their actual L2 achievement and self-perceived proficiency. The results of these linguistic measures were generally comparable to those in (Zhan et al. 2024) whose participants were reported at the lower-intermediate level of L2 proficiency.

**Table 3:** Descriptive statistics of the L2 writing CALF performance.

**Variables**	**Indices**	**Mean**	***SD***	**Min**	**Max**	**Skewness**	**Kurtosis**
Syntactic complexity	MLT	10.36	2.53	5.40	17.80	0.73	0.03
	C/T	1.29	0.19	0.81	2.13	0.96	2.14
	CN/T	1.07	0.44	0.14	2.31	0.50	−0.14
	CP/T	0.20	0.17	0.00	0.89	1.34	2.00
Lexical complexity	LD	0.51	0.04	0.39	0.65	0.17	0.02
	CTTR	4.90	0.71	3.27	6.88	0.22	0.01
	LS2	0.31	0.08	0.13	0.53	0.40	−0.42
Accuracy	EFT/T	0.51	0.18	0.00	0.94	−0.05	−0.19
	EFC/C	0.58	0.17	0.08	0.96	−0.16	−0.22
Fluency	Words	143.21	39.78	46.00	278.00	0.24	0.41
	T-units	14.33	4.50	4.00	31.00	0.81	1.19
	Clauses	18.28	5.73	6.00	37.00	0.64	0.51

### 4.2 Structural equation modeling

The goodness-of-fit indices revealed that the models concerning the relationship between ID factors and L2 writing CALF performance were satisfactory. Table 4 shows the construct validity of the SEM models.

**Table 4:** Construct validity of the SEM models.

**Model**	**Indices**	***x^2^/df***	**CFI**	**TLI**	**SRMR**	**RMSEA**
Syntactic complexity	MLT	1.933	0.952	0.935	0.053	0.050
	C/T	1.939	0.951	0.933	0.053	0.050
	CN/T	1.933	0.951	0.934	0.053	0.050
	CP/T	2.007	0.948	0.929	0.053	0.052
Lexical complexity	LD	1.969	0.950	0.931	0.053	0.051
	CTTR	1.936	0.953	0.936	0.053	0.050
	LS2	1.962	0.950	0.932	0.053	0.051
Accuracy	EFT/T	2.075	0.945	0.925	0.054	0.054
	EFC/C	2.068	0.945	0.925	0.054	0.054
Fluency	W	1.967	0.952	0.934	0.053	0.051
	T	1.971	0.950	0.932	0.053	0.051
	C	1.920	0.952	0.935	0.052	0.050

#### 4.2.1 The direct effects of ID factors on L2 writing CALF performance

The SEM results revealed that L2 writing grit, enjoyment, and working memory did not significantly predict any measure in L2 writing CALF performance, rejecting Hypotheses 1a, 1b, and 1e. L2 writing anxiety significantly predicted L2 writing syntactic complexity and accuracy, but not lexical complexity or fluency, partially supporting Hypothesis 1c. Specifically, L2 writing anxiety served as a significant negative predictor of MLT (β = −0.225, *p* < 0.01), CN/T (β = −0.152, *p* < 0.05), CP/T (β = −0.151, *p* < 0.05), and EFC/C (β = −0.141, *p* < 0.05) with modest effect sizes. These negative coefficients indicated that higher levels of anxiety were associated with producing shorter T-units, fewer complex nominals and coordinate phrases per T-unit, and a lower proportion of error-free clauses. L2 writing anxiety did not significantly predict lexical complexity or accuracy, implying that its debilitating role was more prominent in syntactic structures and form-related precision rather than in vocabulary-related and quantity-related dimensions of performance.

In contrast, L2 writing motivation significantly predicted lexical complexity and fluency, but not syntactic complexity or fluency, partially supporting Hypothesis 1d. Specifically, L2 writing motivation exerted positive effects on CTTR (β = 0.305, *p* < 0.01) with a moderate effect size, and it positively predicted LS2 (β = 0.239, *p* < 0.05) as well as W (β = 0.244, *p* < 0.05) with modest effect sizes. More motivated learners produced texts with greater lexical diversity, used more sophisticated lexical items, and generated a higher number of words within the allotted time. These results further suggested that motivated writers were more inclined to explore lexical choices and sustain writing during the process. Nonetheless, L2 writing motivation did not significantly predict syntactic complexity or accuracy, reflecting the limited cognitive resources available to writers and the competing demands across different CALF dimensions.

#### 4.2.2 The mediating effects of ID factors on L2 writing CALF performance

Although L2 writing grit did not directly predict CALF performance, it exerted indirect effects on L2 writing CALF performance by predicting other ID factors as shown in Table 5. The significant indirect effect sizes of the pathways from L2 writing grit to CALF performance (0.012–0.091) fell within the very small to small range (Fritz and MacKinnon, 2007). Specifically, L2 writing anxiety fully mediated the effect of L2 writing grit on syntactic complexity and accuracy, but the mediating effect of enjoyment on the association between L2 writing grit and CALF performance was not significant, thereby partially supporting Hypothesis 2b and rejecting Hypothesis 2a. Regarding syntactic complexity, L2 writing grit predicted MLT (β = 0.041, 95% CI [0.008, 0.098]), CN/T (β = 0.028, 95% CI [0.001, 0.079]), and CP/T (β = 0.028, 95% CI [0.000, 0.081]) through anxiety. As for accuracy, L2 writing grit predicted EFC/C (β = 0.026, 95% CI [0.003, 0.073]) through anxiety.

**Table 5:** Mediation analysis.

**Outcome**	**Pathway**	**Indirect effect size**	***SE***	**95% CI**	
				**Lower bound**	**Higher bound**
Syntactic complexity	Grit → anxiety → MLT	0.041	0.022	0.008	0.098
	Grit → anxiety → CN/T	0.028	0.019	0.001	0.079
	Grit → anxiety → CP/T	0.028	0.019	0.000	0.081
Lexical complexity	Grit → enjoyment → motivation → CTTR	0.091	0.042	0.032	0.213
	Grit → anxiety → motivation → CTTR	0.016	0.012	0.002	0.053
	Grit → enjoyment → motivation → LS2	0.071	0.045	0.005	0.187
	Grit → anxiety → motivation → LS2	0.012	0.012	0.000	0.049
Accuracy	Grit → anxiety → EFCC	0.026	0.017	0.003	0.073
Fluency	Grit → enjoyment → motivation → W	0.073	0.038	0.020	0.179
	Grit → anxiety → motivation → W	0.012	0.010	0.002	0.046

L2 writing grit significantly predicted lexical complexity and fluency through the serial mediating roles of emotions and motivation, partially supporting Hypotheses 2c and 2e. The pathway from L2 writing grit to CTTR mediated by enjoyment and motivation (β = 95% CI [0.032, 0.213]) accounted for the larger effect size with all the path coefficients being higher than the respective coefficients in the same pathway mediated by anxiety and motivation (β = 0.016, 95% CI [0.002, 0.053]). Specifically, grit exerted a larger effect on enjoyment (β = 0.493, *p* < 0.001) than anxiety (β = −0.185, *p* < 0.05), and the predictive effect of enjoyment (β = 0.603, *p* < 0.001) on motivation was more prominent than that of anxiety (β = −0.276, *p* < 0.001). Similarly, the pathway from L2 writing grit to LS2 mediated by enjoyment and motivation (β = 0.071, 95% CI [0.005, 0.187]) accounted for the larger effect size with all the path coefficients being higher than the respective coefficients in the same pathway mediated by anxiety and motivation (β = 0.012, 95% CI [0.000, 0.049]). Specifically, grit exerted a larger effect on enjoyment (β = 0.493, *p* < 0.001) than anxiety (β = −0.185, *p* < 0.05), and the predictive effect of enjoyment (β = 0.603, *p* < 0.001) on motivation was more prominent than that of anxiety (β = −0.275, *p* < 0.001).

As for fluency, enjoyment and motivation mediated the relationship between grit and W (β = 0.073, 95% CI [0.020, 0.179]). In addition, grit indirectly predicted fluency through the serial mediating roles of anxiety and motivation (β = 0.012, 95% CI [0.002, 0.046]). The pathway from L2 writing grit to W mediated by enjoyment and motivation constituted a larger effect size compared with the same pathway mediated by anxiety and motivation. Specifically, grit exerted a larger effect on enjoyment (β = 0.494, *p* < 0.001) than anxiety (β = −0.185, *p* < 0.05), and motivation was predicted by enjoyment (β = 0.603, *p* < 0.001) to a greater extent than anxiety (β = −0.274, *p* < 0.001). The serial mediating effects of emotions and working memory on the link between L2 writing grit and CALF performance were not significant, notably with the non-significant effects of enjoyment on working memory as well as working memory on L2 writing CALF performance, thereby rejecting Hypotheses 2d and 2f.

Overall, SEM results showed that L2 writing anxiety directly predicted syntactic complexity (i.e., MLT, CN/T, CP/T) and accuracy (i.e., EFC/C), and fully mediated the relationship between grit and syntactic complexity as well as between grit and accuracy. L2 writing motivation directly predicted lexical complexity (i.e., CTTR, LS2) and fluency (i.e., W), and played a crucial role in the serial mediating pathways from L2 writing grit to lexical complexity or fluency. Specifically, L2 writing grit predicted lexical complexity and fluency through enjoyment and motivation as well as through anxiety and motivation.

## 5 Discussion

### 5.1 The direct effects of ID factors on L2 writing CALF performance

The present study extended the application of the CVT (Pekrun, 2006) and the individual-environmental model of writing (Hayes, 1996) to the L2 writing context, and investigated the predictive effects of various ID factors on L2 writing CALF performance. Results revealed that L2 writing anxiety negatively predicted syntactic complexity (i.e., MLT, CN/T, CP/T) and accuracy (i.e., EFC/C), while L2 writing motivation positively predicted lexical complexity (i.e., CTTR, LS2) and fluency (i.e., W). Rather than producing uniform influences across all linguistic outcomes, L2 writing anxiety and motivation selectively affected certain CALF dimensions, consistent with the trade-off hypothesis (Skehan and Foster, 2001), which posits that learners' limited attentional resources necessitate prioritization among competing performance components. Moreover, not all indices within the same CALF dimension were equally susceptible to the effects of anxiety and motivation, indicating that ID factors exert an index-sensitive effect on L2 writing CALF performance.

The negative relationship between L2 writing anxiety and syntactic complexity or accuracy partially corroborated (Zabihi 2018) which showed that anxiety negatively predicted syntactic complexity, accuracy, and fluency. According to the CVT (Pekrun, 2006) and the individual-environmental model of writing (Hayes, 1996), anxiety debilitates cognitive engagement toward the ongoing task and increases task-irrelevant thinking. Additionally, anxious learners generally tend to have avoidance behavior as manifested in the construct of L2 writing anxiety (Cheng, 2004; Guo and Qin, 2010), further resulting in the decrease of the cognitive resources that could be used to complete the writing task. This cognitive strain is particularly evident in influencing syntactic complexity and accuracy which are more cognitively demanding because they require thorough consideration of complex syntactic structures, correct spelling of vocabulary and application of various grammatical rules (Zhan et al., 2024). More specifically, L2 writing anxiety negatively predicted MLT, CN/T and CP/T, as the retrieval of longer T-units and the integration of complex grammatical structures require sustained attention to syntactic planning and coordination of multiple linguistic elements (Vyatkina, 2012). Comparatively, clause-level complexity reflected as C/T emphasizes basic logical connection of clauses rather than the sophistication of modifiers or phrases (Norris and Ortega, 2009). It also requires less conscious planning and attention because clause construction relies more on automatized syntactic routines that are acquired earlier (Biber et al., 2011). As a result, clause embedding tends to be processed more automatically and therefore remains less vulnerable to the attentional disruption caused by anxiety. As for accuracy, L2 writing anxiety significantly predicted EFC/C rather than EFT/T in the present study. Compared with T-level accuracy, clause-level accuracy reflects the correctness of smaller syntactic units (Liu and Shi, 2025), which requires moment-to-moment attentional monitoring, making them sensitive to anxiety-induced attentional disruption. The discrepancy concerning the relationship between anxiety and fluency in (Zabihi 2018) and the present study may be due to the different writing time allocated for task completion. In contrast to the high pressure caused by the limited time of 11 minutes in (Zabihi 2018), the sufficient time provided in the present study (i.e., 30 minutes) enables learners to write more words even in the anxious state. Therefore, fluency may not be influenced by anxiety in the present study.

L2 writing enjoyment did not significantly predict CALF performance in the present study, which contradicts (Abdi Tabari et al. 2024) showing the positive correlation between L2 writing enjoyment and CALF performance. This discrepancy may be due to the inclusion of other interactive factors (e.g., cognitive and motivational factors) in the present study, which disperses the effect of L2 writing enjoyment on CALF performance. The result confirms previous studies demonstrating that interactive factors such as L2 writing motivation (Li et al., 2024) and L2 writing strategies (Zhao et al., 2023) mitigated the influence of enjoyment in L2 writing performance. It could also be explained by the different measurements of enjoyment with trait enjoyment in the present study and task enjoyment in (Abdi Tabari et al. 2024). Task enjoyment is the temporary and context-dependent emotion underlying the ongoing task, which has been revealed to influence the specific task performance to a greater degree than trait enjoyment (Li et al., 2024).

L2 writing motivation positively predicted fluency, which aligns with (Jang and Lee 2019) showing a positive correlation between the ideal L2 self and fluency. As fluency is an important indicator of writing performance and imposes lower demands on processing capacity compared with complexity and accuracy (Fathi and Rahimi, 2022), students with intense writing motivation of gaining a high score in the present study may engage in the more attainable goal, leading them to write sufficient number of words to improve fluency performance. Specifically in the present study, L2 writing motivation significantly predicted the number of words instead of T-units and C-units. This can be explained by the explicit requirement of writing at least 150 words in the task. Highly motivated students can realize the value of the task and endeavor to achieve goals by meeting the target of total number of words, whereas the accumulation of more T-units or clauses is less salient as a goal.

It is worth noting that L2 writing motivation affected lexical complexity but not syntactic complexity in the present study. One possible explanation is that lexis contributes more to the communicative understanding and is then prioritized compared with syntax (Skehan and Foster, 2001). Specifically, learners with high motivation intend to make the content understandable to achieve communicative purpose; therefore, they may direct more attention to lexis. However, the result differs from (Rahimi and Zhang 2019) showing a significant correlation between motivation and syntactic complexity. The inconsistency may be attributed to the participants' different English proficiency levels, with lower-intermediate learners in the present study and upper-intermediate learners in (Rahimi and Zhang 2019). According to (Kim 2021), lower-level L2 learners tend to develop their vocabulary to a greater extent than their syntax, hence motivation is more likely to affect lexical complexity compared to syntactic complexity in this study. And among the three indices of lexical complexity, L2 writing motivation significantly predicted CTTR and LS2 rather than LD. CTTR and LS2 capture learners' use of varied and sophisticated vocabulary, which requires active lexical search and comparison (Lu, 2012). Learns with high motivation, whether intrinsic motivation for exploration or extrinsic motivation for a high score, tend to check a wide lexical repertoire and select advanced forms, consistent with the assumption that motivated writers engage deeply in cognitive processes of text interpretation, reflection, and production (Hayes, 1996). On the contrary, LD refers to the number of content words to total words, which convey information and are selected based on their semantic relevance to the topic (Kisselev et al., 2025), not necessarily because of how motivated the writers feel. Besides, when cognitive resources are directed to complexity, attention to accuracy decreases (Skehan and Foster, 2001). Therefore, motivation does not necessarily guarantee writing accuracy, especially for students who lack L2 competence.

Working memory did not significantly predict any measure of L2 writing CALF performance, which did not align with the CVT and the individual-environmental model of writing assuming its essential role in processing and retrieving relevant information. The null working memory findings may be attributed to its potential interactive effects with other ID factors, particularly learners' L2 writing motivation. Learners in the present study generally reported a high level of L2 writing motivation, which is likely to compensate for their limited cognitive resources by enhancing task engagement, persistence, and strategic effort (Zhang, 2023; Wang et al., 2025). Thus, strong motivational drive may have acted as a buffering mechanism, enabling lower working memory learners to perform comparably to peers with higher working memory. Besides, emotions, particularly anxiety can temporarily reduce the usable working memory resources available to L2 writers (Güvendir and Uzun, 2023), thereby weakening the relationship between working memory and actual linguistic performance. Therefore, the null effects of working memory are likely due not to its theoretical irrelevance but to a combination of motivational compensation and emotional influence that collectively attenuate the observable contribution of working memory to L2 writing CALF outcomes.

### 5.2 The mediating effects of ID factors on L2 writing CALF performance

Although L2 writing grit did not significantly predict CALF performance, it exerted indirect effects on CALF performance. This result provides empirical evidence for the CVT, which assumes that personality factors may have an impact on academic performance via emotions (Pekrun, 2006). Specifically, L2 writing grit predicted syntactic complexity and accuracy via anxiety. Grittier students tend to persist in L2 writing and sustain interest even in face of difficulties and drawbacks, thus experiencing lower anxiety. The current results, together with findings in general L2 context (Teimouri et al., 2022), highlight the role of grit in negatively predicting anxiety in both general L2 and skill-specific L2 learning contexts. Students with a lower level of grit are likely to experience higher anxiety during the writing process, which can impede their cognitive processing of knowledge relevant to the writing task, leading to lower syntactic complexity (Güvendir and Uzun, 2023) and accuracy (Zabihi, 2018).

L2 writing grit influenced lexical complexity and fluency through the serial mediating roles of emotions and motivation, supporting the CVT-based assumption that learners' personality factors influence their emotional experiences, which in turn impact learning motivation and subsequently academic performance (Pekrun, 2006). This is consistent with existing research which showed that grit positively associated with enjoyment and negatively related to anxiety (Teimouri et al., 2022), and emotions significantly linked to motivation (Wang, 2021; Li et al., 2024). Among the serial pathways the present study identified, the sequence in which L2 writing grit predicted lexical complexity or fluency through enjoyment and motivation accounted for larger indirect effects than the sequence involving anxiety and motivation, mainly due to the large effect of enjoyment on motivation. This finding confirms (Dong et al. 2022), which found that enjoyment correlated with motivation to a greater extent than anxiety. According to the CVT, positive emotions (e.g., enjoyment) are expected to enhance both intrinsic and extrinsic motivation, while negative emotions (e.g., anxiety) especially impair intrinsic motivation (Pekrun, 2006). The joint effects of intrinsic and extrinsic motivation aroused by enjoyment may enhance learners' L2 writing lexical complexity and fluency performance to a greater extent than anxiety.

The serial mediating effects of emotions and working memory on the relationship between L2 writing grit and CALF performance were not significant, with non-significant effect of enjoyment on working memory and working memory on CALF performance. Notably, although working memory was negatively predicted by L2 writing anxiety, it was not predicted by L2 writing enjoyment. One possible explanation was that negative emotions (e.g., anxiety) exerted their effect on cognition and behavior in a short time, while the influence of positive emotions (e.g., enjoyment) was shaped over time (Fredrickson, 2001; Dewaele and Alfawzan, 2018). Specifically, when students feel anxious, they tend to instantly direct their attention to task-irrelevant thinking, leaving limited cognitive resources for the writing task and reducing working memory performance. Positive emotions exert their effects on the thought-action repertoire and personal resources (e.g., working memory resources) over a relatively long period of time after accumulated experience, rather than in an immediate way (Fredrickson, 2001). The moderate level of L2 writing enjoyment in the present study may not have reached a threshold necessary to affect working memory.

## 6 Conclusion

The primary focus of the present study is to examine the predictive effects of various ID factors on L2 writing CALF performance. SEM results revealed that L2 writing anxiety negatively predicted syntactic complexity (i.e., MLT, CN/T, CP/T) and accuracy (i.e., EFC/C). L2 writing motivation positively predicted lexical complexity (i.e., CTTR, LS2) and fluency (i.e., W). Although L2 writing grit did not significantly predict writing CALF performance in a direct way, it indirectly predicted syntactic complexity and accuracy through anxiety, and predicted lexical complexity and fluency through the serial mediating roles of emotions and motivation.

There are several limitations of the present study. First, the participants were L2 learners from one high school. Future research could include students from multiple schools with different educational levels and L2 proficiency to enhance the generalizability of the research findings. Second, ID factors were mostly assessed through self-report questionnaires, which may be subject to common method bias as well as potential response biases due to social desirability or inaccurate self-evaluation. Future research could incorporate multiple data sources such as teacher evaluations, reflective journals, interviews, and classroom observations to distinguish construct variance from method-related variance and achieve methodological triangulation. Third, this study adopted questionnaires to measure L2 writing emotions, which largely reflects trait emotions. State emotions (e.g., task emotions) could be further investigated via neuropsychological monitoring or facial expression detection devices, or task emotions questionnaire, allowing for real-time emotion evaluation and comprehensively revealing the role of emotions in L2 writing CALF performance. Furthermore, although the present study revealed the relationship between ID factors and individual CALF measures, future studies could model CALF measures as multiple and interrelated components within a unified framework to obtain a more comprehensive understanding of how ID factors shape L2 writing CALF performance. At last, the present study employed a cross-sectional design and therefore focused only on the unidirectional relationships between ID factors and L2 writing CALF performance. Future research could adopt longitudinal designs to explore reciprocal associations and alternative mediation pathways assumed in the CVT and the individual-environmental model of writing, or use experimental approaches to establish causal relationships.

Despite these limitations, the present study provides theoretical and pedagogical implications. Theoretically, it extends the applicability of the CVT and the individual-environmental model of writing in the L2 writing context, lending empirical support to the structural associations among grit, emotions, motivation, working memory, and L2 writing performance (Hayes, 1996; Pekrun, 2006). The present study particularly uncovers differentiated emotional-motivational pathways for distinct CALF measures, offering a more fine-grained theoretical account of how multiple ID factors interact to shape various aspects of L2 writing CALF performance. By jointly modeling personality, emotional, cognitive, and motivational variables together, the study underscores the value of an integrated perspective in theorizing individual differences in L2 writing. Pedagogically, instructional practices should be aligned with specific learning outcomes. Because anxiety negatively predicts syntactic complexity and accuracy, instruction targeting structurally complex and highly accurate writing should prioritize anxiety-management strategies (e.g., scaffolded planning, supportive feedback), especially in cognitively demanding tasks (e.g., argumentative essays, integrated writing, and decision-making essays). Moreover, as L2 writing motivation positively predicts lexical complexity and fluency, and is more strongly influenced by enjoyment than anxiety, it is necessary to conduct enjoyment-enhancing and motivation-supportive practices (e.g., engaging topics, collaborative writing, autonomy-supportive feedback) to promote more diverse and sophisticated lexical choices and more fluent text production. In addition, teachers should be fully aware of the vital role of L2 writing grit in predicting emotional and motivational ID factors. Although grit is a personality trait, it is malleable through intervention (Shafiee Rad and Jafarpour, 2023). Enhancing learners' grit may foster greater enjoyment, reduce anxiety, and ultimately bolster writing motivation, thereby facilitating L2 writing CALF performance in various aspects. Finally, given that the strength of these pathways may vary by task type, proficiency and learning context, instructional support should be flexibly adapted to learners' needs and situational demands. Overall, the present study advances L2 writing research by elucidating how interconnected ID factors operate through distinct pathways to predict varying CALF measures, providing a more integrated understanding of their roles in L2 writing CALF performance and informing targeted instructional practices.

## Data Availability

The raw data supporting the conclusions of this article will be made available by the authors, without undue reservation.

## Footnotes

1 The direct effects emphasize the pathway from an independent variable to a dependent variable without passing through other variables, while the indirect effects represent how the independent variable predicts the dependent variable through at least one mediator (Hayes, 2018).

2 “Serial mediating effects” refers to the predictive effects of an independent variable on a dependent variable through at least two mediators with one mediator influencing the other (Hayes, 2018).

3 The present study was part of a larger project which compared the predictive effects of ID factors on L2 writing performance at two time points (T1-T2). Three hundred and seventy participants remained at T1, among whom 348 participants further remained at T2. Following Little's (2013) suggestion, the present study adopted multiple imputation to deal with the missing data in longitudinal research.

4 Two topics were selected because the present study was a part of a larger project which collected longitudinal data about writing performance with a counter-balanced design.
