# Supplementary material for: The predictive effects of individual difference factors on L2 writing complexity, accuracy, and fluency
Source: Front Psychol. 2026 Jan 7;16:1631353. doi: 10.3389/fpsyg.2025.1631353 (PMC12819746; doi:10.3389/fpsyg.2025.1631353)
Supplement: Supplementary file 1 [file Data_Sheet_1.pdf]

Supplementary material

L2 writing grit, anxiety, enjoyment and motivation scales

L2 writing grit scale

1. I am a diligent English writing learner.
2. My interests in learning English writing change from year to year.
3. When it comes to English writing, I am a hard-working learner.
4. I think I have lost my interest in learning English writing.
5. Now that I have decided to learn English writing, nothing can prevent me from reaching this goal.
6. I am not as interested in learning English writing as I used to be.
7. I will not allow anything to stop me from my progress in learning English writing.
8. I have been obsessed with learning English writing in the past but later lost interest.
9. I put much time and effort into improving my English writing weaknesses.

L2 writing anxiety scale

1. I feel my heart pounding when I write an English essay with a time limit.
2. When I write an English essay, I feel nervous and uneasy if I know my teacher is going to mark it.
3. I often write what I think and feel in English such as writing diaries and weekly journals in English.
4. I usually avoid writing essays in English whenever possible.
5. Whenever I start to write an essay in English, my mind goes blank.
6. I don’t worry at all that my English essay will be much worse than others.
7. When I write an English essay within a time limit, I tremble with nervousness or break into a cold sweat.
8. I worry about getting a low mark when my English essay is to be corrected.
9. I avoid writing English essays whenever possible.

10. I can’t think clearly when I write an English essay within a time limit.

11. I don’t usually write essays in English unless I have to.

12. I often feel panic when writing an English essay within a time limit.

13. I am worried that other students will laugh at me when they see my English essay.

14. When I am asked to write an English essay within a time limit without any preparation, my brain stops working.

15. I am not worried at all about what other people will say about my English essay.

16. I always look for any possible opportunity to practice English writing outside of class.

17. I feel my whole body becoming stiff and tense when writing an English essay.

18. I am afraid that my English essay will be chosen for class discussion or evaluation.

19. I am not worried at all that my English essay will be scored very low.

20. I write in English whenever I have the chance.

L2 writing enjoyment scale

1. I don’t get bored in English writing.

2. I’ve learnt interesting things during English writing.

3. In class, I feel proud of my accomplishments in English writing.

4. There is a good atmosphere for English writing.

5. Writing in English is fun.

6. The English writing teacher always encourages us to write in English.

7. The English writing teacher is friendly.

8. The English writing teacher is supportive.

9. I enjoy English writing.

10. It’s a positive environment for English writing.

11. We form a tight English writing group.

L2 writing motivation scale

1. I prefer English writing course material that really challenges me so I can learn new things.
2. I prefer English writing course material that arouses my curiosity, even if it is difficult to learn.
3. The most satisfying thing for me in English writing course is trying to understand the content as thoroughly as possible.
4. When I have the opportunity in the English writing class, I choose course assignments that I can learn from even if they don’t guarantee a good grade.
5. Getting a good grade in English writing is satisfying for me.
6. Getting a good grade in English writing is the most important thing for me.
7. If I can, I want to get better grades in English writing than most of the other students.
8. I want to do well in English writing to show my ability to others, like teachers, peers, and family.
